# Parallel implementation of 3D protein structure similarity searches using a GPU and the CUDA

**DOI:** 10.1007/s00894-014-2067-1

**Published:** 2014-01-31

**Authors:** Dariusz Mrozek, Miłosz Brożek, Bożena Małysiak-Mrozek

**Affiliations:** Institute of Informatics, Silesian University of Technology, Gliwice, Poland

**Keywords:** 3D protein structure, Similarity searching, Structure comparison, GPU, CUDA, Parallel programming, Structure matching, Structure alignment

## Abstract

Searching for similar 3D protein structures is one of the primary processes employed in the field of structural bioinformatics. However, the computational complexity of this process means that it is constantly necessary to search for new methods that can perform such a process faster and more efficiently. Finding molecular substructures that complex protein structures have in common is still a challenging task, especially when entire databases containing tens or even hundreds of thousands of protein structures must be scanned. Graphics processing units (GPUs) and general purpose graphics processing units (GPGPUs) can perform many time-consuming and computationally demanding processes much more quickly than a classical CPU can. In this paper, we describe the GPU-based implementation of the CASSERT algorithm for 3D protein structure similarity searching. This algorithm is based on the two-phase alignment of protein structures when matching fragments of the compared proteins. The GPU (GeForce GTX 560Ti: 384 cores, 2GB RAM) implementation of CASSERT (“GPU-CASSERT”) parallelizes both alignment phases and yields an average 180-fold increase in speed over its CPU-based, single-core implementation on an Intel Xeon E5620 (2.40GHz, 4 cores). In this paper, we show that massive parallelization of the 3D structure similarity search process on many-core GPU devices can reduce the execution time of the process, allowing it to be performed in real time. GPU-CASSERT is available at: http://zti.polsl.pl/dmrozek/science/gpucassert/cassert.htm.

## Introduction

Protein 3D structure similarity searching is a process in which a given protein structure is compared to another protein structure or a set of protein structures collected in a database. The aim of the process is to find fragments in common among compared protein structures, i.e., matching fragments. Based on the similarities found during this process, scientists can draw useful conclusions about the common ancestry of the proteins, and thus the organisms (that the proteins came from), their evolutionary relationships, functional similarities, the existence of common functional regions, and many other things [1]. This process is especially important in situations where sequence similarity searches fail or deliver too few clues [2]. There are also other processes in which protein structure similarity searching plays a supportive role, such as in the validation of predicted protein models [3]. Finally, we believe that in the very near future, scientists will have the opportunity to study beautiful structures of proteins taken from a patient in a regular diagnostic procedure that will utilize comparison methods to highlight areas of the proteins that are inadequately constructed, leading to dysfunctions of the body and serious diseases. This goal is currently motivating work leading to the development of similarity searching methods that return results in real time.

Although protein structure similarity searching is one of the primary tasks performed in structural bioinformatics, it is still a very difficult and time-consuming process, mainly because: (1) the 3D structures of proteins are highly complex, (2) the similarity searching process is computationally complex, and (3) the number of 3D structures stored in macromolecular data repositories such as the Protein Data Bank (PDB) [4] is growing exponentially.

Among these three problems, bioinformaticians can attempt to ease the second one by developing new, more efficient algorithms, and to—at least partially—help with the first one by selecting appropriately representative features of protein 3D structures that can then be fed into their algorithms. The collection of algorithms that have been developed for protein structure similarity searching over the last two decades is large, and includes methods such as VAST [5], DALI [6, 7], LOCK2 [8], FATCAT [9], CTSS [10], CE [11], FAST [12], and others [13, 14]. These methods use various representative features when performing protein structure similarity searches in order to reduce the huge search space. For example, local geometric features and selected biological characteristics are used in the CTSS [10] algorithm. Shape signatures that include information on C_α_ atom positions, torsional angles, and types of secondary structure present are calculated for each residue in a protein structure. The DALI algorithm [6, 7], which is well established, compares proteins based on distance matrices built for each of the compared proteins. Each cell of a distance matrix contains the distance between the C_α_ atoms of every pair of residues in the same structure (inter-residue distances). Fragments of 6×6 elements of the matrix are called *contact patterns*, which are compared between two proteins to find the best match. On the other hand, the VAST algorithm [5], which is available through the website of the National Center for Biotechnology Information (NCBI), uses secondary structure elements (SSEs: α-helices and β-sheets), which form the cores of the compared proteins. These SSEs are then mapped to representative vectors, which simplifies the analysis and comparison process. During the comparison, the algorithm attempts to match vectors of pairs of protein structures. Other methods, such as LOCK2 [8], also utilize the SSE representation of protein structure in the comparison process. The CE [11] algorithm uses the combinatorial extension of the alignment path formed by aligned fragment pairs (AFPs). AFPs are fragments of both structures that show clear structural similarity and are described by local geometrical features, including the positions of C_α_ atoms. The idea of AFPs is also used in FATCAT [9].

Even though better methods are developed every year, performing a protein structure similarity search against a whole database of protein 3D structures is still a challenge. As we showed in our previous works [15, 16] on the effectiveness and scalability of the process, performing a search with the FATCAT algorithm for a sample query protein structure using twenty alignment agents working in parallel took 25 hours (without applying any additional acceleration techniques). Tests were carried out using a database containing 3D structures of 106,858 protein chains. This shows how time-consuming the process is, and it is one of the main motivations for designing and developing the new methods that are reported every year, such as RAPIDO [17], DEDAL [18], MICAN [19], CASSERT [13], ClusCo [20], and others [21, 22].

On the other hand, the evolution of computer science and computer architectures has led to (and will continue to lead to) new hardware solutions that can be used to accelerate the 3D structure similarity searches. Recent years have shown that promising results in terms of upscaling the process and accelerating it can be obtained by using graphics processing units (GPUs) and general purpose graphics processing units (GPGPUs). GPU devices, which were originally conceived as a means to render increasingly complex computer graphics, can now be used to perform computations that are required in completely different domains. For this reason, GPU devices, especially those utilizing the NVidia Compute Unified Device Architecture (CUDA) [23, 24], are now widely used to solve computationally intensive problems, including those encountered in bioinformatics. Given the successful applications of GPUs in the fields of sequence similarity [25–31], phylogenetics [32], molecular dynamics [33, 34], and microarray data analysis [35], it is clear that GPU devices are beginning to play a significant role in 3D protein structure similarity searching.

It is worth mentioning two GPU-based implementations of the process. These methods use different representations of protein structures and different computational procedures, but demonstrate a clear improvement in performance over CPU-based implementations. The first one, *SA Tableau Search*, which was first presented in [36], uses simulated annealing for tableau-based protein structure similarity searching. Tableaux are based on orientations of secondary structure elements and distance matrices. The GPU-based implementation of the algorithm parallelizes two areas: multiple iterations of the simulated annealing procedure and multiple comparisons of the query protein structure to many database structures. The second one, called *pssAlign* [37], consists of two alignment phases: *fragment-level alignment* and *residue-level alignment*. Both phases use dynamic programming. In the fragment-level alignment phase, so-called seeds between the target protein and each database protein are used to generate initial alignments. These seeds are represented by the locations of the C_α_ atoms. The initial alignments are then refined in the residue-level alignment phase. pssAlign parallelizes both alignment phases.

In the present paper, we report the GPU-based implementation of the CASSERT algorithm [13] for 3D protein structure similarity searching. Like *pssAlign*, CASSERT is based on two-phase alignment. However, it uses an extended set of structural features to describe protein structures, and the computational procedure differs too. Both the representation of structures and the computational procedure employed by the CASSERT algorithm are presented in the next few subsections. Originally, CASSERT was designed and implemented as a CPU-based procedure, and its effectiveness is reported in [13]. Its GPU-based implementation is presented in the “Methods” section, and this implementation will be referred as “GPU-CASSERT” throughout the paper. Before we start to explain GPU-CASSERT, we will provide a short overview of how to perform computations using GPU devices and the CUDA architecture. Finally, in the “Results” and “Discussion” sections, we show that the algorithm performs fast comparisons of protein structures and that it can be used to scan protein databases in order to find similar biological molecules.

### Representation of protein structures in the comparison process

3D protein structure similarity searching is typically realized by performing pairwise comparisons of the query protein (Q) specified by the user with successive proteins (D) from the database of protein structures. In this section, we show how protein structures are represented in both phases of the comparison process performed by the CASSERT.

Let us assume that* Q* represents the structure of the query protein that is *q* residues (amino acids) long, and* D* is the structure of a candidate protein in the database that is *d* residues (amino acids) long.

In the first phase of the alignment algorithm, protein structures* Q* and* D* are compared by aligning their *reduced chains of secondary structures* consisting of the secondary structure elements *SE*_*i*_:

1 $$ Q=\left(S{E}_1^Q,S{E}_2^Q,\dots, S{E}_n^Q\right), $$

where *n* ≤ *q* is the number of secondary structures in the chain of the query protein* Q*, and

2 $$ D=\left(S{E}_1^D,S{E}_2^D,\dots, S{E}_m^D\right), $$

where *m* ≤ *d* is the number of secondary structures in the chain of the database protein* D*.

**Fig. 1:**
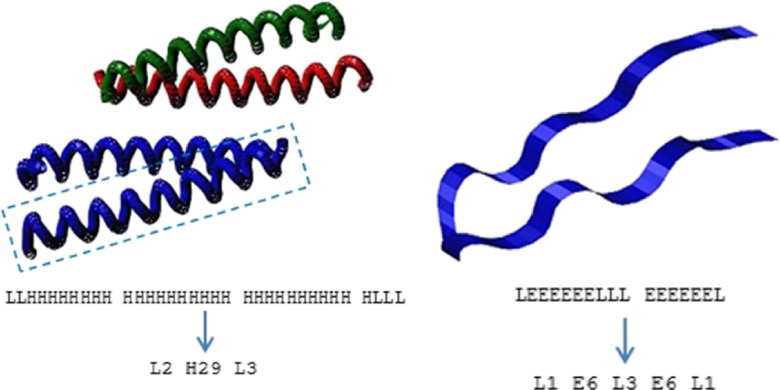
Secondary structure elements: (*left*) four α-helices in a sample structure [PDBID: 1CE9]; (*right*) two β-strands joined by a loop in a sample structure [PDB ID: 1E0Q]; visualized by MViewer [38]. Full and reduced chains of secondary structure elements for the marked subunit (*left*) and the whole structure (*right*) are visible below

Each element *SE*_*i*_, which is a part of the chain that has been selected on the basis of its secondary structure, is characterized by two values, i.e.,

3 $$ S{E}_i=\left[ SS{E}_i,{L}_i\right], $$

where *SSE*_*i*_ describes the type of the secondary structure selected, and *L*_*i*_ is the length of the *i* ^th^ element *SE*_*i*_ (measured in residues). In the alignment method, we distinguish between three basic types of secondary structure (Fig. 1):

- α-Helix (H)
- β-Sheet or β-strand (E)
- Loop, turn, coil, or undetermined structure (L)

Elements *SE*_*i*_^*Q*^ and *SE*_*j*_^*D*^, hereafter referred to as *SE* regions or *SE* fragments, are built from groups of adjacent amino acids that form the same type of secondary structure. For example, six successive residues folded into an α-helix form one *SE* region. Hence, the overall protein structures are, at this stage, represented by the reduced chains of secondary structures.

In the second phase of the alignment algorithm, protein structures* Q* and* D* are represented in more detail. At the residue level, successive residues are described by so-called *molecular residue descriptors s*_*i*_. Proteins are represented as chains of descriptors *s*_*i*_:

4 $$ Q=\left({s}_1^Q,{s}_2^Q,\dots, {s}_q^Q\right), $$

where *q* is the length of the query protein* Q* (i.e., the number of residues it contains), and each *s*_*i*_^*Q*^ corresponds to the *i* ^th^ residue in the chain of protein* Q*,

5 $$ D=\left({s}_1^D,{s}_2^D,\dots, {s}_d^D\right), $$

where *d* is the length of the database protein* D*, and each *s*_*i*_^* D*^ corresponds to the *i* ^th^ residue in the chain of protein* D*.

**Fig. 2:**
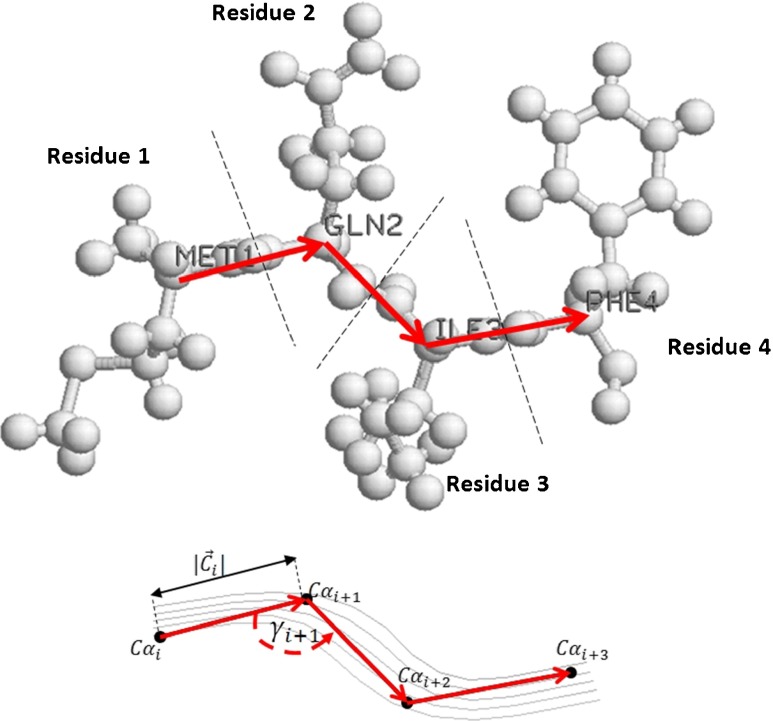
Structural features included in molecular residue descriptors marked on part of a sample protein structure: residue type (Met, Gln, Ile, Phe), secondary structure type (β-strand in this case), length of the vector between the C_α_ atoms (|*C*_*i*_|), and the *γ* angle

Each descriptor *s*_*i*_ is defined by the following vector of features:

6 $$ {s}_i=<\left|{C}_i\right|,{\gamma}_i, SS{E}_i,{r}_i>, $$

where |*C*_*i*_| is the length of the vector between the C_α_ atoms of the *i* ^th^ and (*i* + 1)^th^ amino acids in a protein chain, *γ*_*i*_ is the angle between the successive vectors *C*_*i*_ and *C*_*i* + 1_, *SSE*_*i*_ is the type of secondary structure formed by the *i* ^th^ residue, and *r*_*i*_ is the type of amino acid represented by this residue (Fig. 2).

### General course of the matching method

**Fig. 3:**
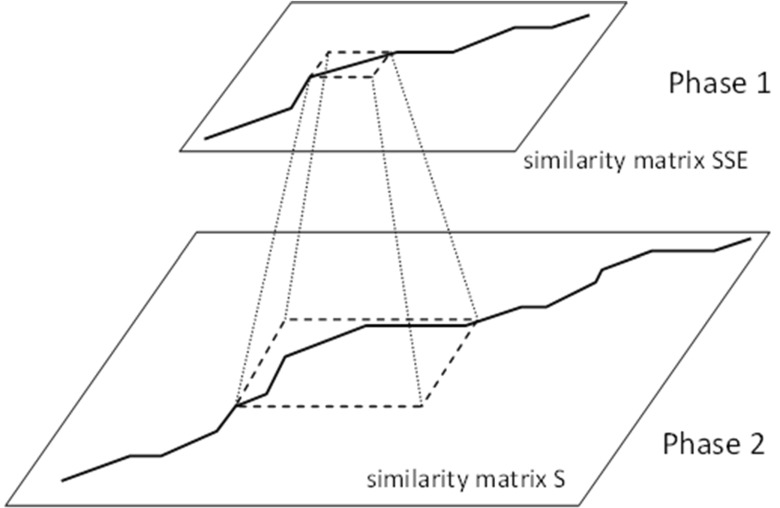
Overview of the two-phase alignment algorithm. In phase 1, low-resolution alignment is performed: protein structures are represented as reduced chains of secondary structures; the similarity matrix* SSE* used in the alignment is small—proportional to the number of secondary structures in both proteins. In phase 2, high-resolution alignment is performed: protein structures are represented as chains of molecular residue descriptors; the similarity matrix* S *used in the alignment is therefore large—proportional to the length of both proteins

Pairwise comparisons of protein 3D structures are performed using the matching method, which consists of two phases (Fig. 3):

1. The first phase involves the coarse alignment of spatial structures represented by secondary structure elements (SSEs). This is the *low-resolution alignment* phase, because groups of amino acids occurring in each structure are grouped into one representative element (the *SE* region). This phase allows us to run fast alignments in which small similarity matrices are constructed. This eliminates the need for computationally costly alignments of proteins that are entirely dissimilar. Proteins that exhibit secondary structure similarity are subjected to a more thorough analysis in the second phase.
2. The second phase involvs the detailed alignment of spatial structures represented by the molecular residue descriptors. This alignment is performed based on the results of the coarse alignment realized in the first phase. The second phase is the *high-resolution alignment* phase, because amino acids are not grouped in it. Instead, each amino acid found in the structure is represented by the corresponding molecular residue descriptor *s*_*i*_. Therefore, we align sequences of molecular residue descriptors using much larger similarity matrices than were utilized in the first phase. In the second phase, the algorithm analyzes more features describing protein structures, and the protein itself is represented in more detail.

In both phases, the alignments are carried out using dynamic programming procedures that are specifically adapted to the molecular descriptions of protein structures employed in each phase. The detailed courses of both alignment phases are shown in the following subsections.

### First phase: low-resolution alignment

The low-resolution alignment phase is performed in order to filter out molecules that do not show secondary structural similarity. Originally, this phase was also used to establish initial alignments that were projected onto the similarity matrix in the second phase. However, since both phases are executed independently in the GPU-based implementation, we do not transfer alignment paths between alignment phases in the GPU-based approach.

In order to match the structures of proteins* Q* and* D* that are represented as reduced chains of secondary structures, we build a similarity matrix* SSE* of size *n* × *m*, where *n* and *m* describe the number of secondary structures in the compared chains of proteins* Q *and* D*. Successive cells of the SSE matrix are filled according to the following rules:

For 0 ≤ *i* ≤ *n* and 0 ≤ *j* ≤ *m*:

7 $$ SS{E}_{i,0}= SS{E}_{0,j}=0, $$

8 $$ SS{E}_{i,j}^{(1)}= SS{E}_{i-1,j-1}+{\delta}_{ij}, $$

9 $$ SS{E}_{i,j}^{(2)}={E}_{i,j}, $$

10 $$ SS{E}_{i,j}^{(3)}={F}_{i,j}, $$

11 $$ SS{E}_{i,j}=\underset{v=1..3}{ \max}\left\{ SS{E}_{i,j}^{(v)},0\right\}. $$

where *δ*_*ij*_ is the similarity reward, which reflects the degree of similarity between two regions *SE*_*i*_^*Q*^ and *SE*_*j*_^*D*^ of proteins* Q* and* D*, respectively, and vectors* E* and* F* are possible horizontal and vertical penalties for inserting a gap.

The similarity reward *δ*_*ij*_ takes values in the interval [0,1], where 0 means no similarity and 1 means that the regions are identical. The degree of similarity is calculated using the formula

12 $$ {\delta}_{ij}={\sigma}_{ij}-\left({\sigma}_{ij}\frac{\left|{L}_j^D-{L}_i^Q\right|}{\left({L}_j^D+{L}_i^Q\right)}\right), $$

where *L*_*i*_^*Q*^, *L*_*j*_^*D*^ are the lengths of the compared regions *SE*_*i*_^*Q*^ and *SE*_*j*_^*D*^, while *σ*_*ij*_ describes the degree of similarity of the secondary structures for the *i* ^th^ and *j* ^th^ *SE* regions of the compared proteins* Q* and* D*. This parameter can take three possible values, according to the following rules:

(i) *σ*_*ij*_ = 1 when both *SE* regions have the same α-helix or β-strand structure
(ii) *σ*_*ij*_ = 0.5 when at least one of the regions is a loop, turn, coil, or its secondary structure is undefined
(iii) *σ*_*ij*_ = 0 when one of the regions is an α-helix and the second is a β-strand

Values of gap penalty vectors are calculated as follows:

13 $$ {E}_{i,j}= \max \left\{\begin{array}{c}\hfill {E}_{i,j-1}-{g}_E\hfill \\ {}\hfill SS{E}_{i,j-1}-{g}_O\hfill \end{array}\right., $$

14 $$ {F}_{i,j}= \max \left\{\begin{array}{c}\hfill {F}_{i-1,j}-{g}_E\hfill \\ {}\hfill SS{E}_{i-1,j}-{g}_O\hfill \end{array}\right.. $$

In order to assess the similarity between two reduced chains of secondary structures, we use the *Score* measure, which is equal to the highest value in the similarity matrix SSE:

15 $$ Score= \max \left\{ SS{E}_{i,j}\right\}. $$

Auxiliary vectors* E* and* F* allow us to perform the alignment procedure and to calculate the *Score* similarity measure in linear space, because the value of cell *SSE*_*i*,*j*_ depends only on the value of cell *SSE*_*i* − 1,*j* − 1_, *SSE*_*i* − 1,*j*_, and *SSE*_*i*,*j* − 1_. During the calculation of the similarity matrix* SSE*, we must store the position of the maximum value of the *Score* in the matrix as well as the value itself.

### Second phase: high-resolution alignment

Molecules that pass the first phase (based on the user-defined cutoff value) are further aligned in the second phase. A database protein structure qualifies for the second phase if the following condition is satisfied:

16 $$ \frac{ Scor{e}^{QD}}{ Scor{e}^{QQ}}\ge {Q}_t, $$

where *Score* ^*QD*^ is a similarity measure employed when matching the query protein structure to the database protein structure, *Score* ^*QQ*^ is the similarity measure obtained when matching the query protein structure to itself (i.e., the maximum *Score* that the compared chain can achieve), and *Q*_*t*_ ∈ [0,1] is a user-defined qualification threshold for structural similarity.

The second phase is carried out similarly to the first phase, except that the alignment is carried out at the residue level, where aligned molecules* Q* and* D* are represented by chains of molecular residue descriptors. However, the way that GPU-CASSERT calculates the similarity reward for the two compared residue molecular descriptors *s*_*i*_ and *s*_*j*_ is different. The similarity reward *ss*_*ij*_ is calculated according to the following formula:

17 $$ s{s}_{ij}={w}_C{\sigma}_{ij}^C+{w}_{\gamma }{\sigma}_{ij}^{\gamma }+{w}_{SSE}{\sigma}_{ij}^{SSE}+{w}_{\mathrm{r}}{\sigma}_{ij}^{\mathrm{r}}, $$

where *σ*_*ij*_^*C*^ is the degree of similarity of a pair of vectors *C*_*i*_^*Q*^ and *C*_*j*_^*D*^ in proteins* Q* and* D*, respectively, *σ*_*ij*_^*γ*^ is the similarity of angles *γ*_*i*_^*Q*^ and *γ*_*j*_^*D*^ in proteins* Q* and* D*, *σ*_*ij*_^*SSE*^ is the degree of similarity of the secondary structures of residues *i* and *j* (calculated according to rules (i)–(iii) listed for the first phase), *σ*_*ij*_^r^ is the degree of similarity of the residues defined by means of the BLOSUM62 substitution matrix normalized to the range [0,1], and *w*_*C*_, *w*_*γ*_, *w*_*SSE*_, and *w*_r_ are the weights of all of the components (with default values of 1).

The similarity of vectors *C*_*i*_^*Q*^ and *C*_*j*_^*D*^ is defined according to the formula:

18 $$ {\sigma}_{ij}^C={\mathrm{e}}^{-{\displaystyle {\left(\left|{C}_i^Q\left|-\right|{C}_j^D\right|\right)}^2}}, $$

where |*C*_*i*_^*Q*^| and |*C*_*j*_^*D*^| are the lengths of vectors *C*_*i*_^*Q*^ and *C*_*j*_^*D*^, respectively, and the similarity of the angles *γ*_*i*_^*Q*^ and *γ*_*j*_^*D*^ is defined as follows:

19 $$ {\sigma}_{ij}^{\gamma }={\mathrm{e}}^{-{\displaystyle {\left({\gamma}_i^Q-{\gamma}_j^D\right)}^2}}. $$

In high-resolution alignment, the value of the degree of similarity of molecular residue descriptors *ss*_*ij*_ (Eq. 17) replaces the similarity reward *δ*_*ij*_ (Eq. 8).

The relative strength of each component in the similarity search (Eq. 17) can be controlled using participation weights. The default value for each is 1, but this can be changed by the user. For example, researchers who are looking for surprising structural similarities but no sequence similarity can disable the component for the primary structure by setting the value of *w*_*r*_ = 0.

The *Score* similarity measure, a basic measure of the similarity of protein structures, is calculated in this phase. This value incorporates all possible rewards for a match, mismatch penalties, and penalties for inserting gaps into the alignment. The *Score* is also used to rank highly similar proteins that are returned by the GPU-CASSERT.

### Third phase: structural superposition and alignment visualization

In the third phase, we perform superposition of protein structures on the basis of aligned chains of molecular residue descriptors. The purpose of this step is to match two protein structures by performing a set of rotation and translation operations that minimize the RMSD:

20 $$ RMSD=\sqrt{\frac{1}{N}{\displaystyle \sum_{i=1}^N{d}_i^2}}, $$

where *N* is the number of aligned C_α_ atoms in the protein backbones, and *d*_*i*_ is the distance between the *i* ^th^ pair of atoms. We use the Kabsch algorithm [39] to complete this step. However, the calculation is performed on the CPU of the host workstation.

In this phase, we also calculate the full similarity matrix* S* in order to allow backtracking from the maximum value and full visualization of the structural alignment at the residue level. This step is performed on the CPU of the host and only for a limited number (*M*, which is configured by the user) of the most similar molecules.

## Methods

Greatly accelerated calculation speeds are possible with GPUs, but this also necessitates the application of an appropriate programming model. Before we begin describing the GPU-based implementation of the CASSERT algorithm, we now describe some operational details of GPU devices and the CUDA architecture. These details are an important aid to understanding our implementation.

### The CUDA architecture and the construction of GPU devices

**Fig. 4:**
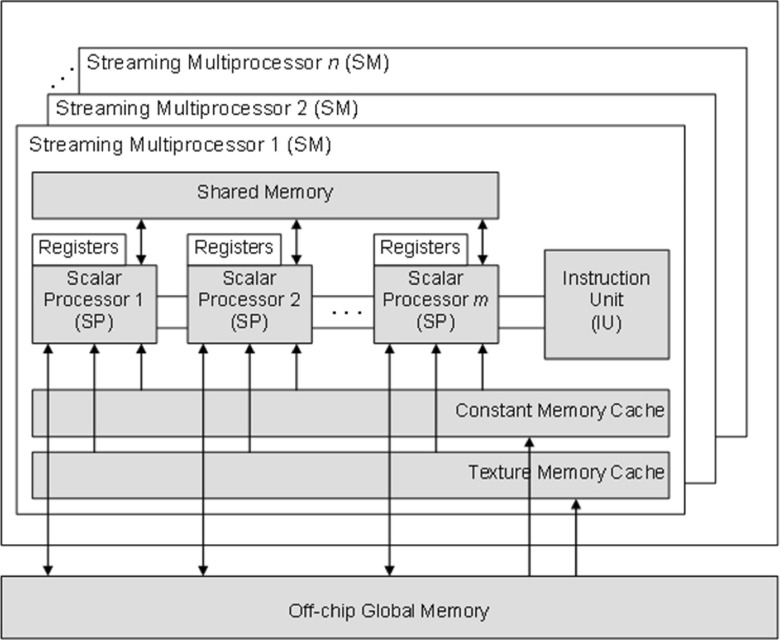
Architecture of the GPU computing device, showing streaming multiprocessors, scalar processor cores, registers, and global, shared, constant, and texture memories

In GPU devices that support the CUDA architecture, high scalability is achieved by the hierarchical organization of *threads*, which are basic execution units. Threads execute, in parallel, user-defined procedures called *kernels*, which implement some computational logic that is applied to data. Each thread has its own index, the vector of the coordinates corresponding to its location in the one-, two-, or three-dimensional organizational structure called a *block*. Thread blocks form a one- or two-dimensional structure called a *grid*. Each thread block is processed by a streaming multiprocessor (SM), which has many scalar processor cores (SP). The number of multiprocessors and processor cores available depends on the type of GPU device used. The GPU device has also two special function units, a multithreaded instruction unit (IU), a set of registers available for each thread block, and several types of memory (Fig. 4).

Threads can access global memory, which is the off-chip memory that has a relatively low bandwidth but provides a high storage capacity. Each thread also has access to the on-chip read–write shared memory as well as the read-only constant memory and texture memory, both of which are cached on-chip. Access to these three types of memories is much faster than that to the global memory, but they all provide limited storage space and are used in specific situations.

Multiprocessors employ a new architecture, called SIMT (single instruction, multiple thread). In this architecture, a multiprocessor maps each thread to a scalar processor core, where each thread executes independently with its own instruction address and register state. The multiprocessor SIMT unit creates, manages, schedules, and executes threads in groups of 32 parallel threads called *warps*. Threads in the warp perform the same instructions, but operate on different data, as in the SIMD (single instruction multiple data) architecture. Therefore, appropriate preparation and arrangement of data is highly desirable before the kernel execution begins, and this is one of the factors that influence the efficiency of any GPU-based implementation [23].

### Data preparation

Early tests of the first implementations of the CASSERT algorithm on GPU devices showed that read operations from the database system storing structural data were too slow. Therefore, the present implementation of the GPU-CASSERT does not read data directly from the database, because single execution of the searching procedure would take too long. We have introduced binary files instead. These files contain data packages that are ready to be sent to the GPU device. The only data that are read directly from the database are those that describe the query protein structure* Q*. But, even in this situation, the data are stored in a appropriate way in binary files. Using binary files with data packages allows the initialization time of the GPU device to be reduced severalfold. This is necessary to ensure that GPU-CASSERT has a fast response time.

Binary files must be refreshed in two cases:

- Changes in the content of a database
- Changes in parameters affecting the construction of data packages

Data packages that are sent to the GPU device have the same general structure, regardless of what is stored inside.

Due to the size of the data packages utilized by the CASSERT algorithm, these packages are placed in the global memory of the GPU device. As we know from the “The CUDA architecture and the construction of GPU devices,” when discussed GPUs and the CUDA, global memory is the slowest type of memory available. For this reason, it is worth minimizing the number of accesses made of this type of memory.

**Fig. 5:**
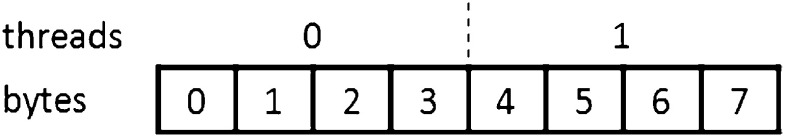
Preferred distribution method for the first 8 bytes of the transaction among threads. Thread 0 takes the first 4 bytes of the transaction, thread 1 takes the next 4 bytes, etc.

Access operations are carried out in 32-, 64-, or 128-byte transactions. When the warp (which is composed of 32 threads) reaches the read/write operation, the GPU device attempts to perform this operation using a minimum number of transactions. Basically, the greater the number of transactions needed, the greater the amount of unnecessary data transmitted. This unnecessary overhead can be minimized for CUDA 2.x if memory cells that are read by all warp threads are located within a single 128-byte memory segment. In order to satisfy this condition, the address of this area must be aligned to 128 bytes and the threads need to read data from adjacent memory cells. For devices with compute capabilities of 1.0 or 1.1, upon which GPU-CASSERT can also run, there is the additional restriction that warp threads must be in the same order as the memory cells being read [23]. If these conditions are met, we can get 4 bytes of data for each of the threads in a single 128-byte transaction. These 4 bytes correspond to a single number of type *int* or *float*. The preferred distribution method for the first 8 bytes of the transaction among threads is presented in Fig. 5. The remaining bytes of the transaction should be distributed in the same way.

**Fig. 6:**
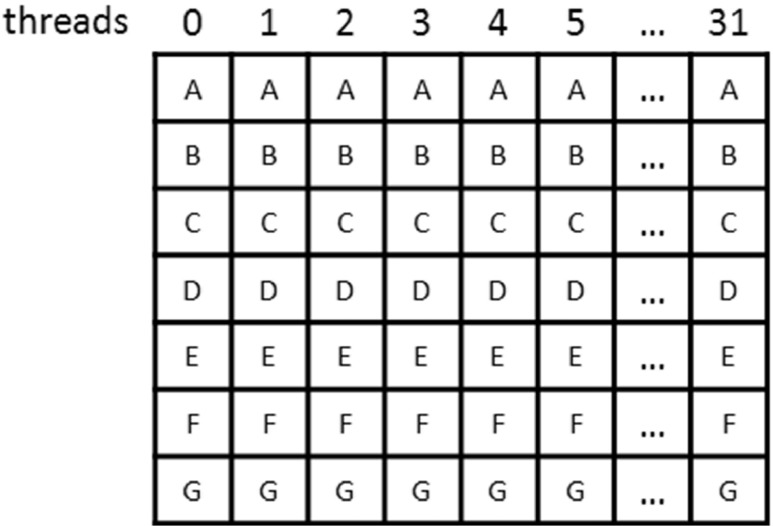
Arrangement of chains of structural descriptors in a memory array. Block threads are assigned to particular columns. Each cell contains 4 bytes of data (structural descriptors). All block threads read contiguous memory areas

Data are transmitted to the GPU device in the form of a two-dimensional array of unsigned integers (Fig. 6). The array is organized in row-major order. This means that the cells in adjacent columns are located next to each other in the memory. This has an important influence on performance when processing an array, because contiguous array cells can usually be accessed more quickly than cells that are not contiguous. Each column of the array is assigned to a single block thread. A single chain of structural descriptors is stored in a single column of the array (Fig. 6). Such a solution satisfies the condition that contiguous addresses must be read, because block threads will always read adjacent cells, moving from the beginning to the end of the chain (from top to bottom). Every cell in the array is 4 bytes in size, so the transfer of data to a wrap’s 32 threads will be made in one 128-byte read transaction. This allows us to take a full advantage of data transfer from the memory to the registers of the GPU device. This way of organizing data in memory is used and described in [28, 29].

Another factor affecting the performance is the density at which the data are packed in memory cells. The distribution of data in memory cells depends on the phase of the algorithm and the type of structural descriptors that are used in the phase. There are five types of data that are sent to the memory of the GPU device:

- Reduced chains of secondary structures formed by secondary structure elements *SE*_*i*_ (phase 1)
- Secondary structure elements *SSE*_*i*_ that are components of nonreduced chains of molecular residue descriptors (phase 2)
- Amino acid residue types *r*_*i*_ that are components of nonreduced chains of molecular residue descriptors (phase 2)
- Lengths of the vectors between C_α_ atoms of subsequent residues that are components of nonreduced chains of molecular residue descriptors (phase 2)
- *γ*_*i*_ angles between successive vectors *C*_*i*_ and *C*_*i* + 1_ that are components of nonreduced chains of molecular residue descriptors (phase 2)

Regardless of the type of data present in the memory cells, the chains included in the package may be of various lengths. For this reason, all chains of structural descriptors are aligned to the length of the longest chain. Empty cells are filled with zeros. In principle, comparing these zeros during the course of the algorithm does not affect the scoring system assumed and the final results.

Chains of structural descriptors contained in a data package are sorted by their lengths in ascending order. In this way, we minimize differences in processing time for individual block threads and their idle times (threads that have already completed their work must wait for the other threads to finish processing). A similar method is used in the work presented in [28, 29].

Data packages are divided into subpackages. Each subpackage consists of 32 chains of structural descriptors. This is exactly the same as the number of warp threads.

### Implementation of two-phase structural alignment in a GPU

Implementation of the two-phase structural alignment algorithm in a GPU with the CUDA requires a dedicated approach. GPU-CASSERT operates according to the following scheme:

1. Read data packages describing database protein structures from binary files
2. Read query protein structure (*Q*) from database and create appropriate data packages
3. Perform the first phase of the structural alignment on the GPU device for all query protein (*Q*) vs. database protein (*D*) pairs
4. Perform the second phase of the pairwise structural alignment for the molecules that passed the first phase (based on the given threshold) on the GPU device
5. Return a list of the top *M* database molecules that are most similar to the query molecule, together with similarity measures
6. If the user wants to visualize the alignment, perform the second phase on the CPU of the host computer for molecules from the list of the most similar ones to the query molecule returned by the GPU device
7. Return alignment visualization to the user

In both alignment phases, the vector of penalties for a gap and the similarity matrix are stored in the global memory of the GPU device as arrays of type *float*. This means that a read/write of a single element requires just one transaction. It is also worth noting that, due to memory restrictions, each thread remembers only the last row of the similarity matrix. This is sufficient to determine the maximum element of the similarity matrix, which also provides a value for the *Score* similarity measure, which is needed to check whether a database structure qualifies for the second phase. The similarity measure alone is sufficient to assess the quality of the alignment before the second phase. On the other hand, the second phase is performed on the GPU device for all qualified structures, and once again on the CPU of the host for the database proteins that are most similar to the query molecule in order to get alignment paths and to perform structural superposition. As a result, we obtain a list of the structures that match most closely to the query structure and a visualization of the local alignments of these structures at the residue level.

### First phase of structural alignment in the GPU

**Fig. 7:**
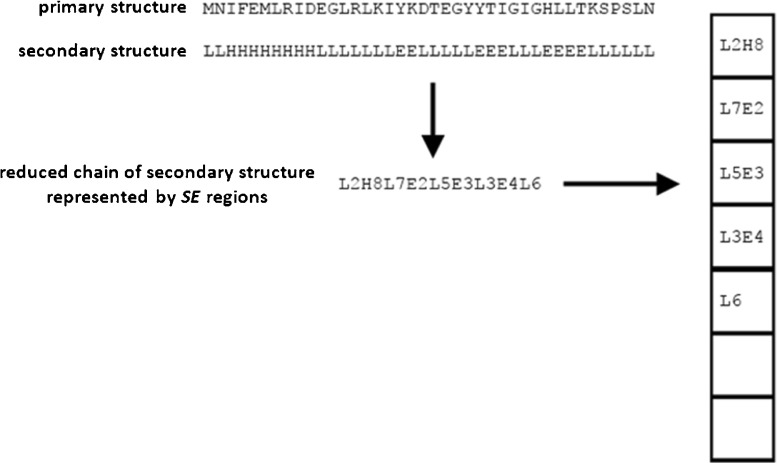
Encoding of a reduced chain of secondary structure in a data package. The secondary structure of the protein is first translated into a reduced chain of* SE* regions. Subsequently, every two* SE *regions are placed in a data package in the manner shown, taking up 4 bytes, and in such a way that they are loaded into the global memory of the GPU device

The first phase requires data to be delivered in the form of data packages containing reduced chains of secondary structures (*SE* regions). Separate data packages are built for the query protein and candidate protein structures from the database. For the purpose of processing, *SE* regions are encoded using two bytes: one byte for the type of secondary structure and one byte for its length. Types of secondary structures are mapped to integers. In the “Data preparation,” where we described the overall structure of a data package, we also mentioned that the data in memory are arranged in 4-byte cells. In such a 4-byte cell we can store two encoded *SE* regions. This is illustrated in Fig. 7.

**Fig. 8:**
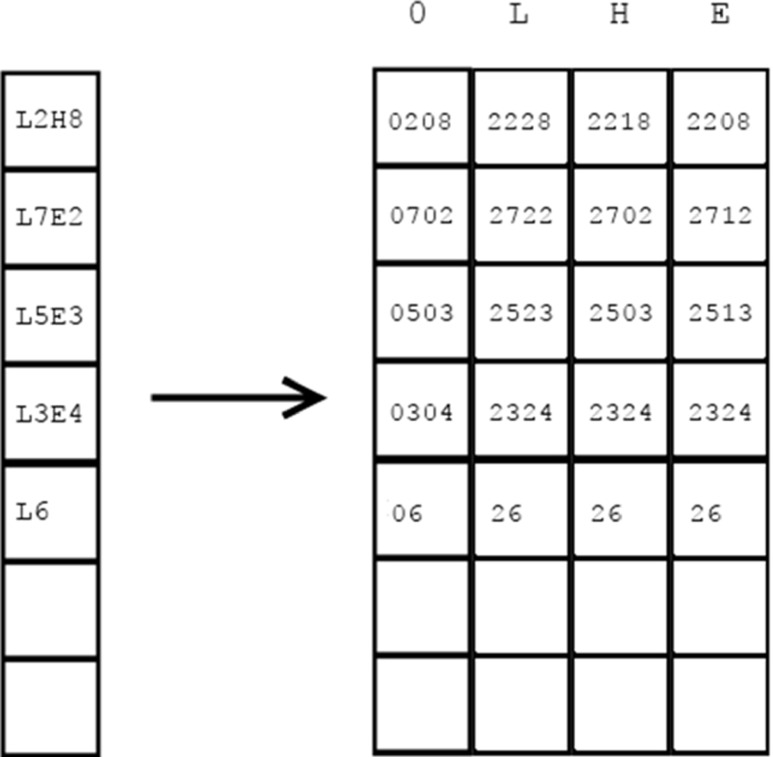
Encoding the reduced chain of secondary structure for query protein* Q* (*left*) and constructing the query profile (*right*). The query profile shows all possible (encoded) scores when comparing the reduced query chain of secondary structure to* SE* regions from candidate protein structures from the database

The data package for the query chain of secondary structures is built on the basis of a slightly different principle. If it was created in the same way as the data packages for database structures, then in order to extract the similarity coefficient of secondary structures *σ*_*i*,*j*_ we would have to read the cell (*SSE*_*i*_^*A*^, *SSE*_*j*_^*B*^) from a predefined matrix of coefficients (a kind of substitution matrix constructed based on rules (i)–(iii) in the “Introduction” section), which would affect performance negatively. We can avoid this by pre-computing and writing all possible similarity coefficients directly into the data package of the query protein, creating something like the query-specific substitution matrix proposed in [40] and called a *query profile* in the GPU-based alignment algorithm for sequence similarity presented in [28]. Therefore, the data package for the query protein passes through an additional preparation step. For each *SE* region, four versions of the similarity coefficient are created, one for each of the secondary structure types and one for the neutral element 0 (as shown in Fig. 8). In the query profile created, the row index is defined by the index of the structural region* SE* divided by 2, and the column index is defined by the type of secondary structure present (with the additional neutral element 0). The coefficients are converted to integers in order to fit them into 1 byte, according to the following rules:

- If coefficient *σ*_*i*,*j*_ = 0, it is encoded as 0
- If coefficient *σ*_*i*,*j*_ = 1, it is encoded as 1
- If coefficient *σ*_*i*,*j*_ = 0.5, it is encoded as 2

The lengths of *SE* regions do not change. This process is illustrated in Fig. 8.

Once the data packages are loaded into the host memory and a data package for the reduced query chain is created, the program transfers data to the GPU device. To do this, it uses four streams. Each stream has its own memory buffers on the GPU device side and in the page-locked memory on the host side. The host loads data into the page-locked memory and then initiates asynchronous data transfer to GPU device for each of the streams. This allows transmission to take place in parallel with the ongoing calculations, again improving performance. Results are received prior to the transfer of the next data package or after all available packages have been processed.

**Fig. 9:**
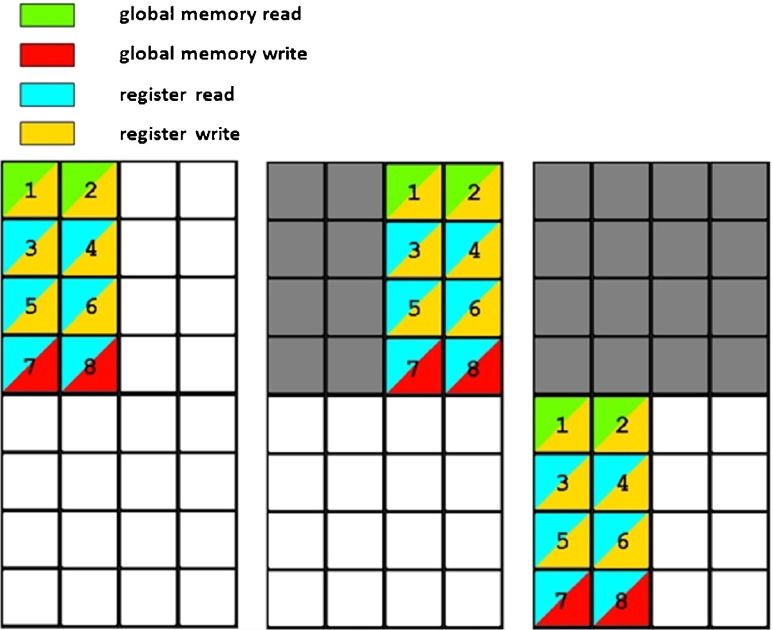
Calculation of the similarity matrix* SSE*. Structural elements (*SE* regions) of the candidate database structure are (virtually) located along the vertical edge of the matrix and* SE* regions of the query protein structure along the horizontal edge of the matrix. Calculations are performed in areas 2×4 in size. Values of the cells in these areas are calculated according to the given order.* Colors *reflect the type of read/write operation required and the memory resources that are affected

Block threads perform parallel alignments of reduced chains of secondary structures. Each block thread performs a pairwise alignment of the query protein vs. one candidate database protein. In order to limit the number of accesses of the global memory of the GPU device, the similarity matrix* SSE* is not calculated cell by cell but is divided into rectangular *areas* of size 2×4. Calculations are performed area by area, and row by row in each area, from left to right, as shown in Fig. 9.

Structural elements (*SE* regions) of the candidate database structure are (virtually) located along the vertical edge of the matrix, and *SE* regions of the query protein structure are located along the horizontal edge of the matrix. During the calculation of each 2×4 area, the values of the four elements of the vector* E* representing the vertical gap penalty and structural data for the four elements of the database chain are stored in GPU registers. Calculation of a 2×4 area requires two reads and two writes to the global memory for the vector* F* representing the horizontal gap penalty, and two reads and two writes for the similarity matrix* SSE*. It also requires four reads for the query profile placed in the texture memory. In total, the calculation of 8 cells of an area of the similarity matrix* SSE* requires eight read/write transactions to the global memory of the GPU device and four reads from the texture memory. The order of calculation of cells and the read/write operations performed are shown in Fig. 9.

After filtering candidate database proteins based on the qualification threshold *Q*_T_, the program creates new, smaller data packages that are needed in the second phase.

### Second phase of structural alignment in the GPU

In the second phase, separate data packages are built for each of the features included in the molecular residue descriptors. In data packages for amino acid types and secondary structure types, we can store elements for four successive molecular residue descriptors in every 4 bytes (and then in every 4-byte memory cell). The arrangement of bytes and cells in memory is similar to that used in the first phase. Vector lengths and angles occupy 4 bytes each, which is one cell of the prepared array in memory.

For the query protein structure, data packages for amino acid types and secondary structures are generated in a similar manner to how this is done in the first phase. The program creates separate query profiles for secondary structures and for residue types. The query profile for secondary structures is formed from the secondary structure similarity coefficients *σ*_*i*,*j*_ in such a way that the row index is the index of the current element from the query chain divided by 4, and the column index is the type of the secondary structure of the element from the compared database protein (Fig. 8). The query profile for residue types is derived from the normalized BLOSUM substitution matrix in such a way that the row index is the index of the current element from the query chain divided by 4, and the column index is the type of the residue from the compared database chain. Data packages containing vector lengths and angles between these vectors, for the query protein structure, are created by rewriting these values to separate packages.

**Fig. 10:**
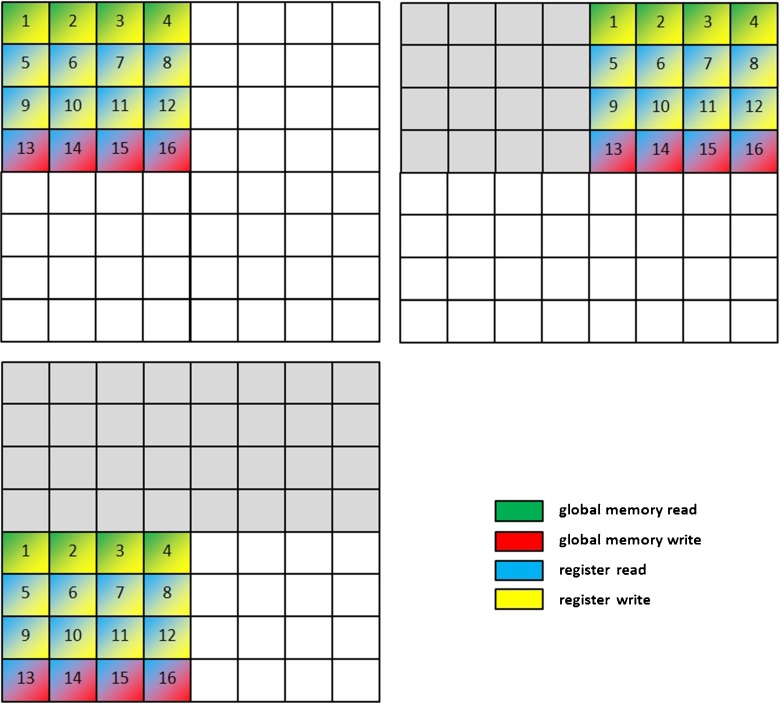
Calculation of the similarity matrix* S* in the second phase of alignment. Molecular residue descriptors of the candidate database structure are (virtually) located along the vertical edge of the matrix and molecular residue descriptors of the query protein structure are located along the horizontal edge of the matrix. Calculations are performed in areas of size 4×4. Values of the cells in these areas are calculated according to the given order.* Colors* reflect the type of read/write operation that are required and the memory resources that are affected

Transfer of data packages to the device is performed in the same manner as in the first phase. Four streams are used for this purpose. After the first part of the data has been transferred to the GPU device, the high-resolution alignment procedure is initiated. Block threads perform parallel alignments of chains of molecular residue descriptors. Each block thread performs a pairwise alignment of the query protein vs. one candidate database protein. In order to limit the number of accesses to the global memory of the GPU device, the similarity matrix* S* is divided into rectangular areas of size 4 × 4. Calculations are performed area by-area, and row by row inside each area, from left to right, as shown in Fig. 10.

Molecular residue descriptors of the candidate database structure are (virtually) located along the left vertical edge of the matrix* S*, and molecular residue descriptors of the query protein structure are located along the top horizontal edge of the matrix. During the calculation of each 4 × 4 area, the values of the four elements of the vector* E* representing the vertical gap penalty and the molecular residue descriptors for four successive elements of the database chain are stored in GPU registers. Calculation of a 4×4 area requires four reads and four writes to the global memory for the vector* F* representing the horizontal gap penalty, and four reads and four writes for the similarity matrix* S*. It is also necessary to perform four reads for the query profile for secondary structures, four reads for the query profile for residue types, four reads for vector lengths, and four reads for angles between vectors. These reads are performed from the texture memory, where these structural features are placed and arranged in an appropriate manner. In total, the calculation of the 16 cells in each area of the similarity matrix* S* requires 16 read/write transactions to the global memory of the GPU device and 16 reads from the texture memory. The order of calculation of cells and the read/write operations performed are shown in Fig. 10.

## Results

We have tested the efficiency of the GPU-CASSERT algorithm and compared it with the CPU-based implementation that was published in [13]. Both implementations, i.e., the GPU-based and the CPU-based implementations, were tested on a Lenovo ThinkStation D20 with two Intel Xeon CPU E5620 2.4 GHz processors, 16 GB of RAM, and a GeForce GTX 560 Ti graphics card with 2GB of GDDR5 memory. The workstation had the Microsoft Windows Server 2008 R2 Datacenter 64-bit operating system installed, together with the CUDA SDK version 4.2. The CUDA compute capability supported by the graphics card was 2.1. The graphics card had the following features:

- 8 streaming multiprocessors (384 processing cores)
- 48 KB of shared memory per block
- 64 KB of total constant memory
- 32,768 registers per block
- 2 GB of total global memory

Tests were conducted using the DALI database (the same as that used by the DALI algorithm [6, 7]), which contained the structures for 105,580 protein chains. While testing performance, we used 14 selected query protein structures with lengths between 29 and 2005 amino acids. These were randomly selected molecules that represent different classes according to the SCOP classification [41], i.e., all α, all β, α + β, α/β, α and β, coiled coil proteins, and others. The list of query protein structures used in the tests performed in the present work is shown in Table 1.

**Table 1:** Query protein structures used in the performance tests

PDB ID	Chain	Length	PDB ID	Chain	Length
2CCE	A	29	1AYE	_	400
2A2B	A	40	2EPO	B	600
1BE3	G	80	1KK7	A	802
1A1A	B	101	1URJ	A	1027
1AYY	B	142	2PDA	A	1230
2RAS	A	199	2R93	A	1421
1TA3	B	300	2PFF	B	2005

**Fig. 11:**
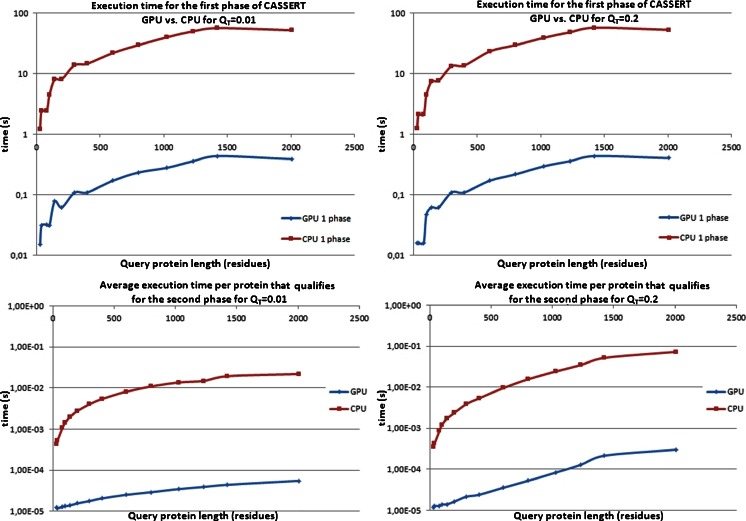
Total execution time for the first phase (*top*) and average execution time of both phases per protein that qualified for the second phase (*bottom*) for qualification thresholds of 0.01 (*left*) and 0.2 (*right*) as a function of the length of the query protein structure* Q*. Time is plotted on a log_10_ scale. Comparison of two implementations of the CASSERT algorithm: CPU-based (*red*) and GPU-based (CUDA,* blue*). Results for 14 selected query protein structures between 29 and 2005 amino acids long. Searches were performed against the DALI database, containing 105,580 structures

Tests were performed using different qualification thresholds (*Q*_T_ = 0.01, 0.2, 0.4, 0.6, 0.8) that the structures had to attain for them to pass from the first phase to the second phase of CASSERT. CASSERT execution times for *Q*_T_ = 0.01 and *Q*_T_ = 0.2 are shown in Fig. 11. The thresholds used were not chosen randomly. *Q*_T_ = 0.2 is an experimentally determined threshold that filters out a reasonable number of structures based on secondary structure similarity but still allows short local similarities to be found. This will be discussed further later in the section. *Q*_T_ = 0.01 means that almost no filtering is done based on the secondary structure similarity, and almost all structures in the database qualify for the second phase.

The results of the efficiency tests presented in Fig. 11 prove that GPU-CASSERT scans the database much faster than the CPU-based implementation. Upon analyzing execution times for the first phase of the CASSERT algorithm (Fig. 11 top) for both qualification thresholds (top left and top right), we can see that increasing the query protein’s length causes the execution time for the algorithm to increase too. This is expected, since a longer query protein chain implies a longer alignment time for every pair of compared proteins. Small fluctuations that are visible for short chains when using the GPU-based implementation and *Q*_T_ = 0.01 (top left, blue) are caused by variations in the number of secondary structures identified in the investigated proteins, which affect the alignment time. We observe a similar (expected) dependency between the length of the query protein and the execution time while analyzing the measured execution times after both phases of the CASSERT algorithm for both qualification thresholds (Fig. 11, bottom left and bottom right). However, since the number of proteins that qualify for the second phase varies and depends on the length and complexity of the query structure, we show average execution times per qualified protein in Fig. 11 (bottom). We noticed that, in some cases, more database protein structures qualified for the second phase for shorter rather than longer (between 1000 and 2000 residues) query protein structures.

**Fig. 12:**
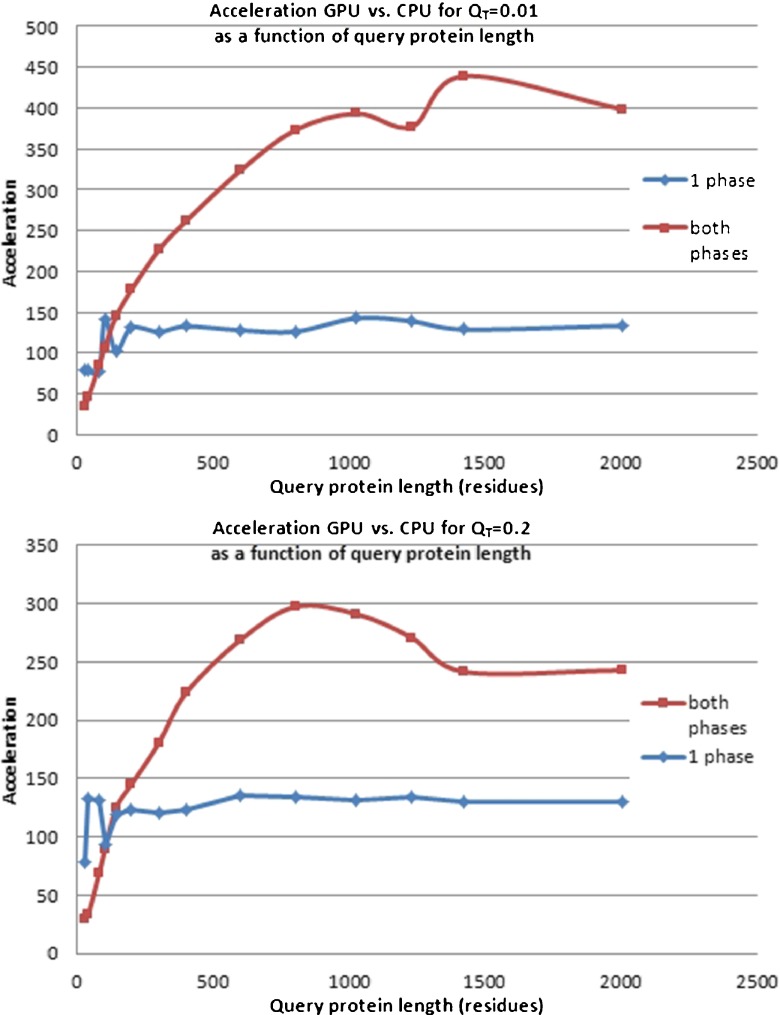
Acceleration achieved by GPU-CASSERT with respect to CPU-CASSERT as a function of query protein length after the first phase (*blue*) and both alignment phases (*red*) with qualification thresholds of 0.01 (*top*) and 0.2 (*bottom*)

Using the execution time measurements that we have obtained during the performance tests, we also calculated acceleration ratios for GPU-CASSERT with respect to CPU-CASSERT. Figure 12 shows how the acceleration ratio changes as a function of query protein length for the first phase and both phases for *Q*_T_ = 0.01 (top) and *Q*_*T*_ = 0.2 (bottom).

We can see that the acceleration ratio for the first phase remains stable. In this phase, GPU-CASSERT is on average 120 times faster than CPU-CASSERT. However, for the whole alignment, i.e., after the first and second phases, the acceleration ratio greatly depends on the length of the query protein structure, its construction and complexity. The whole alignment process when performed on the GPU is 30–300 times faster than the same process performed on the CPU.

Actually, for qualification thresholds *Q*_T_ ≥ 0.1, we observed a kind of compensation effect. For longer query protein chains, which also have more complicated constructions in terms of secondary structure, the number of candidate structures from the database that qualified for the second phase decreases with the length of the query protein. This causes a situation in which fewer database proteins need to be aligned during the entire process. But, at the same time, the length of the query protein grows, causing the alignment time to increase. This growth is compensated for by the smaller number of database structures that need to be aligned.

**Fig. 13:**
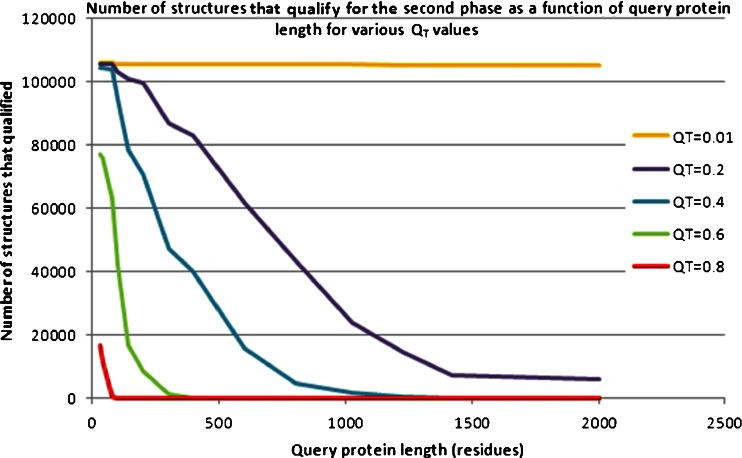
Number of structures from the database that qualified for the second phase as a function of query protein length for various values of the qualification threshold

Figure 13 shows the relationship between query protein length and the number of structures that qualified for the second phase when various values of the qualification threshold *Q*_T_ were applied. For example, for *Q*_T_ = 0.01 (yellow line), we can see that almost all of the database structures qualified for the second phase, regardless of query protein length. In this cases, there is practically no filtering based on the secondary structures identified in the query protein. On the other hand, for *Q*_T_ = 0.8 (red line), we noticed that for query proteins over 150 residues in length, only single database structures are eligible for further processing.

In many situations, such a high value of the qualification threshold will filter out too many molecules. However, this depends on the situation for which the entire process of similarity searching is carried out. For example, in homology modeling, we may want to find referential protein structures that are very similar to the given query protein structure. For functional annotation and while searching for homologous structures, *Q*_T_ = 0.2 could be a reasonable threshold, since it filters out many candidate molecules and, even for very long query proteins, it allows several thousands of structures at least to pass through to the second phase.

We should also remember that the first alignment phase can be turned off completely by specifying *Q*_T_ = 0.0. Then, all of the database molecules pass through to the second phase, which prolongs the similarity searching process.

## Discussion

The results of the efficiency tests we performed have confirmed our expectations. Using a graphics card with a CUDA compute capability is one of the most efficient approaches to use when performing protein structure similarity searching. Upon comparing execution times, we can see that the GPU-based implementation is several dozen to several hundred times faster (an average of 180 times faster for *Q*_T_ = 0.2) than the CPU-based implementation. This is very important, since the number of protein structures in macromolecular databases, such as the Protein Data Bank, is growing very quickly, and the dynamics of this growth is also increasing. The use of GPU-based implementations is particularly convenient for such processes because GPU devices are reasonably inexpensive compared to, say, big computer clusters. Our experiments were performed on a middle-class GPU device, which was set up on a small PC workstation with two processors. For this reason, GPU devices can be usefully applied in the implementation of many algorithms in the field of bioinformatics.

The novelty of CPU-CASSSERT lies mainly in the fast preselection phase based on secondary structures (the low-resolution alignment phase), which precedes the phase of detailed alignment (the high-resolution alignment phase). This allows the number of structures that will be processed in the second, costly phase to be limited, which, in turn, significantly accelerates the method itself. A comparison of CPU-CASSERT with the popular DALI and FATCAT algorithms is presented in [13].

GPU-CASSERT provides additional acceleration over its CPU-based version by executing the computational procedure in parallel threads on multiple cores of the GPU device. The resulting increase in speed is even greater than those achieved with the methods mentioned in the “Introduction” of this paper. SA Tableau Search provides a 33-fold increase in speed when using a GTX 285 graphics card and a 24-fold increase when using a C1060 GPU device rather than the CPU implementation. However, the optimization procedure is based on simulated annealing, which is run in parallel CUDA threads. Individual thread blocks perform the optimization procedure for different candidate protein structures from a database. Protein structures are represented as tableaux containing the orientations of secondary structure elements and distance matrices. However, one of the problems with this algorithm is encountered when comparing big protein structures that generate big tableaux and distance matrices, as they cannot be stored inside the constant and shared memory during computations. This makes it necessary to use a slower version of the GPU kernel which exploits the global memory rather than the faster constant and shared memory. GPU-CASSERT avoids this problem by using a different representation of protein structures: linear sequences of structural descriptors (where secondary structure elements are also included) are employed rather than two-dimensional representative structures.

In terms of representation of protein structures and the implementation of the method, GPU-CASSERT is closer to pssAlign [37], which shows up to a 35-fold increase in speed with the NVIDIA Tesla C2050 GPU over its CPU-based implementation. Both algorithms consists of two alignment phases. The fragment-level alignment phase of pssAlign uses an index-based matched fragment set (MFS) in order to find so-called seeds between the target protein and each database protein. These seeds, which are represented by the locations of the C_α_ atoms, are used to generate initial alignments which are then refined in the residue-level alignment phase. Just like GPU-CASSERT, both phases utilize dynamic programming. However, in GPU-CASSERT, the low-resolution alignment phase is treated as a preselection phase for detailed alignment. In contrast to pssAlign, both phases are executed independently in GPU-CASSERT. We do not store alignment paths after the first phase of the algorithm, which was done in the original CASSERT published in [13]. Consequently, we also do not perform backtracking in the kernel of the first phase, since GPU-CASSERT only needs the *Score* measure to calculate the qualification threshold *Q*_T_ for the next phase. The *Score* is calculated in a linear space, which also influences the effectiveness. Backtracking is also not performed in the GPU after the high-resolution alignment phase. It is executed on the host instead, and only for the highest-scoring database molecules that are returned for the user to visualize. This allows computational time to be saved.

Additional savings can be achieved when working with small query structures. After filtering candidate database proteins based on the qualification threshold, the program creates new, smaller data packages that are needed in the second phase. This usually takes some time. For this reason, for shorter query proteins (less than 100 amino acids in length), it is reasonable to omit the first phase by setting the qualification threshold to 0.0. The probability that such a small protein structure (after it has been reduced to a chain of *SE* regions) will be similar to many of the database proteins is very high. This means that all or almost all of the proteins qualify for the next phase (this is visible in Fig. 13), which makes the first preselection phase almost useless.

GPU-CASSERT also provides additional unique features. Following research into GPU-based sequence alignments [25, 26, 28, 29], we arrange the data in an appropriate manner before sending them to the global memory of the GPU device. Chains of structural descriptors representing protein structures are stored in a prepared memory array that guarantees coalesced access to the global memory in a single transaction. Structural descriptors are not transferred to the global memory of the GPU device directly from a database, but they are stored in binary files, which enables faster transfer, and they are sorted by their lengths in order to reduce thread idle time once they are processed. We also encoded secondary structure descriptors of query protein structures (in both phases) as query profiles—appropriate matrices of all possible scores. During the computations performed on the GPU device, the query profile and substitution matrix (needed in the second phase) are located in the texture memory. The texture memory is cached on the chip of the graphics card and provides a higher effective bandwidth, reducing the number of requests made to off-chip global memory. Streaming is also applied in GPU-CASSERT in order to alternate kernel launches and memory copies, resulting in further acceleration. Finally, we optimized the kernel code to avoid introducing branching via conditional statements.

GPU-CASSERT is available at: http://zti.polsl.pl/dmrozek/science/gpucassert/cassert.htm

## Summary

Efficient methods of 3D protein structure similarity searching are required, as well as their new, efficient implementations, in order to generate results in a reasonable time, considering the exponentially growing numbers of protein structures in macromolecular repositories. In this paper, we have presented GPU-CASSERT, a GPU-based implementation of the CASSERT algorithm for efficiently scanning a database of protein structures in order to identify structural similarities.

As noted in this paper and the papers of other researchers, at the current stage of development of computer science, GPU devices provide an excellent alternative to very expensive computer infrastructures, as they allow large increases in speed over CPU-based implementations for the same computational methods. Moreover, taking into account that the number of processing cores and the amount of memory in modern GPU devices are constantly growing, the computational capabilities of GPU devices are also growing at the same time. Although, implementing computational methods requires some additional effort by the user, including the need to get familiar with the completely new CUDA architecture and programming model, and to refactor the code of existing procedures into GPU kernels, in return we can achieve much faster processing. This is very important because, for many processes such as 3D protein structure similarity searching, reducing computational complexity is a very difficult, if not impossible, task. GPU-based implementations like that presented in the present paper do not reduce the complexity, but they can speed up the process by implementing massive parallelization, thus reducing the overall time required for process execution.
